# Whole Genome Analysis Revealed the Genes Responsible for Citreoviridin Biosynthesis in *Penicillium citreonigrum*

**DOI:** 10.3390/toxins12020125

**Published:** 2020-02-15

**Authors:** Takumi Okano, Naoki Kobayashi, Kazuki Izawa, Tomoya Yoshinari, Yoshiko Sugita-Konishi

**Affiliations:** 1 Graduate School of Life and Environmental Sciences, Azabu University, Kanagawa 252-5201, Japan; 2 Department of Computer Science, Tokyo Institute of Technology, Tokyo 152-8550, Japan; 3 Division of Microbiology, National Institute of Health and Sciences, Kanagawa 210-9501, Japan

**Keywords:** biosynthesis gene cluster, citreoviridin, draft genome, *Penicillium citreonigrum*

## Abstract

Citreoviridin (CTV) is a mycotoxin that is produced by *Aspergillus terreus*, *Eupenicillium ochrosalmoneum* and *Penicillium citreonigrum*, and CTV has been detected in a wide range of cereal grains throughout the world. Furthermore, it is especially a serious problem in regions where rice is consumed as a staple food. Moreover, CTV is a well-known yellow rice toxin, and outbreaks of beriberi have occurred due to consumption of rice that is contaminated by CTV even in the recent years. Although CTV biosynthetic genes of *A. terreus* have been described, those of *P. citreonigrum* remain unclear, which is concerning since *P. citreonigrum* is the main cause of CTV contamination in rice. In the present study, we determined the draft genome of the *P. citreonigrum* strain IMI92228 and revealed the presence of all four genes that form a gene cluster and that are homologous to the CTV biosynthesis genes of *A. terreus*. The expression of these four homologous genes were highly correlated with CTV production, suggesting that they may play an important role in CTV biosynthesis in *P. citreonigrum*. We concluded that the gene cluster is a CTV biosynthesis cluster of *P. citreonigrum*. The findings will contribute to the understanding of the biosynthetic pathway of CTV and will ultimately lead to improvements in the CTV management of agricultural products.

## 1. Introduction

Citreoviridin (CTV) is one of the mycotoxins that contaminates a wide range of agricultural products, including rice. It is produced by *Aspergillus terreus*, *Eupenicillium ochrosalmoneum* and *Penicillium citreonigrum* [1,2,3]. Toxicological studies indicate that CTV could induce acute neurotoxic effects, such as respiratory failure, circulatory failure, paralysis and convulsions, in humans and experimental animals [4,5,6]. Previous studies demonstrate the relationship between CTV and several human diseases. Several decades ago, shoshin-kakke (cardiac beriberi) occurred in Japan, and it is related to the consumption of molded yellow rice, which is contaminated with CTV produced by *P. citreonigrum* [7]. Recently, outbreaks of beriberi have occurred in Brazil, and *P. citreonigrum* and CTV were detected in rice samples of related outbreaks [8,9]. CTV has also been implicated in Keshan disease, which is an endemic cardiomyopathy condition of China and North Korea, through an oxidative stress mechanism [10]. Additionally, it is possible that CTV is a risk factor for the development of atherosclerosis [11].

CTV is a highly reduced polyketide product, and its chemical structure was determined in the 1960s by Sakabe [12]. The structure is similar to aurovertins, which are potent inhibitors of mitochondrial respiration, and in vitro studies demonstrate that CTV inhibits triphosphate adenosine and thiamine diphosphate, which may reveal a potential mechanism for how CTV causes cardiac beriberi [13,14,15].

Recently, the gene cluster for CTV biosynthesis was identified in *A. terreus* ver. *aureus* [16]. Lin et al. revealed the cluster by a resistance-gene-driven genome mining method. To achieve self-resistance against its self-produced metabolites, fungi may occasionally harbor duplicated resistant targets within the biosynthetic gene cluster [17,18]. As such, an extra gene copy of the F1-ATPase β-subunit, a well-known target of CTV, was found and named *ctvE*. Furthermore, *ctvE* was located next to putative enzyme genes that are involved in the synthesis of CTV. A highly reducing polyketide synthase (HR-PKS) gene (*ctvA*), and the following genes for other putative enzymes are required to synthesize the tetrahydrofuran ring and methylated α-pyrone in CTV: methyltransferase (*ctvB*), flavin-dependent mono-oxygenase (*ctvC*) and hydrolase (*ctvD*). Heterologous expression in *A. nidulans* revealed that these four genes together are sufficient for CTV formation [16]. Nevertheless, the gene cluster for CTV biosynthesis in *P. citreonigrum*, which is a species that is believed to be responsible for yellow rice toxicity and recent outbreaks of beriberi, has still been unclear. In the present study, we hypothesized that homologous genes to the CTV biosynthesis genes in *A. terreus* are involved in the biosynthesis of CTV in *P. citreonigrum*, and as such, these genes are searched for in the genome.

## 2. Results

### 2.1. Whole Genome Sequence

To investigate whether *P. citreonigrum* has homologous genes to the CTV biosynthesis genes in *A. terreus*, we conducted a whole genome shotgun analysis using the *P. citreonigrum* strain IMI92228. In total, 76,116,858 paired-end raw reads were obtained. After quality filtering, 37,808,090 forward reads and 32,889,633 reverse reads remained. 32,731,394 reads had pairs and 5,076,696 reads were single. After the assemble and scaffolding, we obtained 79 scaffolds that were more than 500 bp each and approximately 27 Mbp in total length (Table 1).

**Figure 1:**
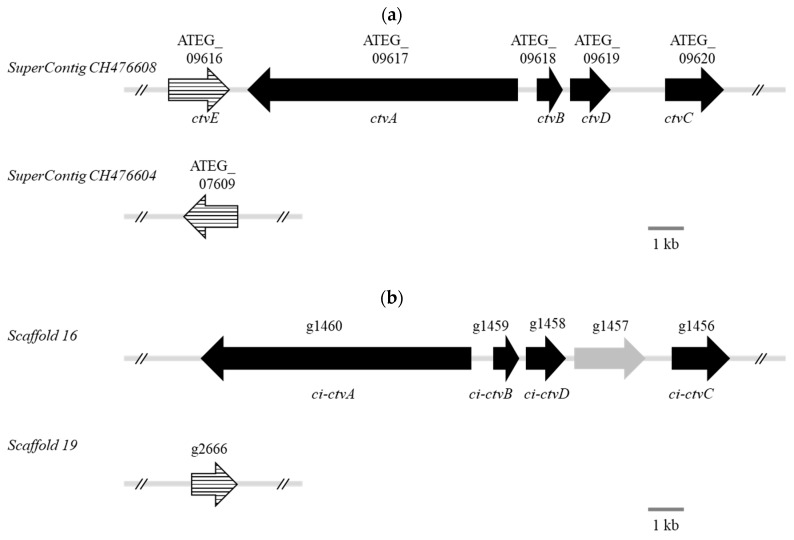
The gene clusters for citreoviridin biosynthesis in *Aspergillus terreus* (**a**) and *Penicillium citreonigrum* (**b**). Black arrows indicate the genes of the enzymes involved in citreoviridin biosynthesis. Striped arrows indicate the gene for the F1-ATPase β-subunit. Gray arrow indicates a gene for the putative transporter. Scale bar indicates 1 kb.

From the open reading frame (ORF) prediction, 9805 ORFs were predicted, and analysis of single copy genes conserved in *Eurotiomycetes* showed that 97.3% (3936 genes out of 4046 genes) of genes were found in the obtained scaffolds. The estimated complete genome size was approximately 28 Mbp (27,997,905 bp). All the predicted ORFs were subjected to alignment using protein basic local alignment search tool (BLASTP) with the following five genes that are involved in the CTV biosynthesis of *A. terreus* and which form a gene cluster in *A. terreus* genome: *ctvA*, *ctvB*, *ctvC*, *ctvD* and *ctvE* (Figure 1a). As a result, we found that the predicted ORFs in the *P. citreonigrum* strain IMI92228 genome showed high homology to all five CTV biosynthesis genes in *A. terreus* (Table 2). Among these, the ORFs that showed high homology to *ctvA*, *ctvB*, *ctvC* and *ctvD* were located on scaffold 16 (2,344,991 bp) (Table 2 and Figure 1). We considered that these specific predicted ORFs were homologous to *ctvA* to *ctvD* in *A. terreus* and were named *ci-ctvA* to *ci-ctvD* (Accession number: LC517105 and LC517107 to LC517109). These predicted genes were also found to be arranged in the same order and direction as that of *A. terreus*, although there is a gene (g1457, LC517106) that cannot be found in *A. terreus*, between *ci-ctvC* and *ci-ctvD* (Figure 1b). The gene g1457 is similar to HC-toxin efflux carrier TOXA of *Talaromyces islandicus*. Meanwhile, the ORF that showed high homology to *ctvE* was separated from the others and located on scaffold 19 (2,964,612 bp) (Table 2 and Figure 1). There was only one ORF (g2666, LC517110) that showed high homology to *ctvE* in the *P. citreonigrum* genome and the ORF showed higher identity to one of the duplicated genes of the F1-ATPase β-subunit in *A. terreus*, other than *ctvE* (ATEG_07609, identity = 90.0%).

### 2.2. Citreoviridin Production Profile

**Figure 2:**
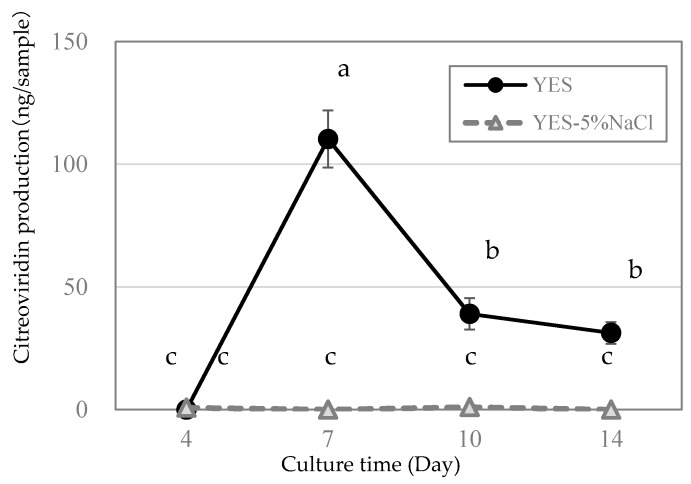
Citreoviridin production by the *Penicillium citreonigrum*, strain IMI92228. *Penicillium citreonigrum* was cultivated on YES agar (black circle) and YES agar with 5% NaCl (gray triangle) at 25 °C. Error bars represent the standard error of three replicates. Different letters indicate statistically significant differences (*p* < 0.05).

*P. citreonigrum* was cultured on YES agar and YES agar with 5% NaCl, and CTV production was monitored for 2 weeks. CTV production on the YES agar peaked on day 7 (110.3 ± 11.7 ng/sample) and decreased on day 10 (Figure 2). CTV production was suppressed at a value lower than 1.1 ± 0.4 ng/sample on YES agar with 5% NaCl.

### 2.3. Gene Expression

**Figure 3:**
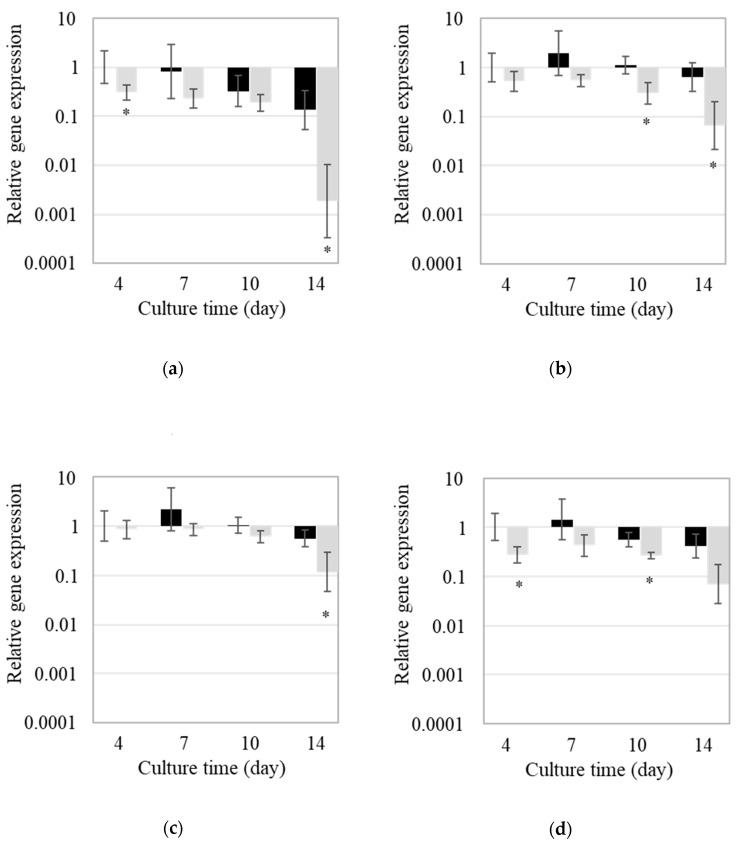
Gene expression profile of the citreoviridin biosynthesis genes of *Penicillium citreonigrum* during citreoviridin production: (**a**) *ci-ctvA*, (**b**) *ci-ctvB*, (**c**) *ci-ctvC*, (**d**) *ci-ctvD*. The x-axis denotes culture time (day) and the y-axis indicates the relative expression level of each gene. Black bars represent the expression of the culture on YES agar and gray bars represents that on YES agar with 5% NaCl. Error bars represent the standard error of three replicates. Asterisks indicate statistically significant differences between cultures on YES agar and YES agar with 5% NaCl (*p* < 0.05).

To validate whether the homologous genes to the CTV biosynthesis genes of *A. terreus* are involved in CTV biosynthesis in *P. citreonigrum*, a gene expression analysis of these genes was carried out during 14-day cultures on YES agar and YES with 5% NaCl agar. The expression of *ci-ctvA* was highest at day 4 and then gradually decreased on YES agar (Figure 3a). The expressions of *ci-ctvB*, *ci-ctvC* and *ci-ctvD* peaked on day 7 (Figure 3b–d). On YES agar with 5% NaCl, the expression of *ci-ctvA*, *ci-ctvB*, *ci-ctvC* and *ci-ctvD* tended to decrease compared to those of YES agar.

## 3. Discussion

The genes responsible for CTV biosynthesis were previously identified in *A. terreus* ver. *aureus* [16], while those of *P. citreonigrum*, which is an important species that causes most of the world’s CTV contamination of rice, are veiled. Thus, in the present study, we aimed to identify the genes for CTV biosynthesis in the *P. citreonigrum* genome.

Biosynthesis of fungal secondary metabolites, including mycotoxins, are generally produced through numerous enzymatic steps [19,20]. The genes that encode the enzymes to produce the secondary metabolites are generally located near each other and form a biosynthetic gene cluster on the chromosome. For example, biosynthesis of aflatoxin, which is the most studied and well-known mycotoxin as a highly toxic and carcinogenic secondary metabolite, requires 28 genes to be produced, and these genes are clustered on chromosome 3 in *A. flavus* [21,22,23]. The genes for CTV biosynthesis in *A. terreus* also form a cluster (Figure 1a) [16]. In the present study, we determined the draft genome of *P. citreonigrum* strain IMI92228 and searched for genes that are homologous to the biosynthesis genes of CTV in *A. terreus*.

Next-generation sequencing of the *P. citreonigrum* strain IMI92228 yielded a total of 7611 Mbp, and the final assembly was found to contain 27 Mbp in 79 scaffolds. The total length of scaffolds and estimated genome size were relatively small compared to previously reported genomes of the *Penicillium* species [24,25,26,27], although these scaffolds code 97.3% of single copy genes that were conserved in *Eurotiomycetes*, which is enough to search for the homologous gene cluster that corresponds to the CTV biosynthetic cluster of *A. terreus*. The estimated genome size may be underestimated because only short inset sizes were estimated, and short read analysis may be insufficient to evaluate repeat regions and transposable elements of the genome. Further analysis on the genome structure of *P. citreonigrum* strain IMI92228 will provide more accurate information on genome size. Furthermore, we found genes that show high homology to all four CTV biosynthesis genes in *A. terreus*-*ctvA* to *ctvD*, and which are sufficient for CTV formation (Table 2). We named these genes *ci-ctvA* to *ci-ctvD.* These four predicted genes were located near each other on scaffold 16 and form a gene cluster, such as that in the *A. terreus* genome (Figure 1). Interestingly, the direction and order were also similar to those in the *A. terreus* genome except for the presence of an extra gene, g1457, between *ci-ctvC* and *ci-ctvD*, and which is not found in the *A. terreus* genome. Generally, in the biosynthetic gene clusters for the same secondary metabolite, the order of the homologous genes differs among species, which suggests that the precise gene order is not crucial for function. For example, the gene order markedly differs between the aflatoxin biosynthetic gene cluster in *A. flavus* and the sterigmatocystin cluster in *A. nidulans*, although *A. flavus* also produces sterigmatocystin as an intermediate of aflatoxin [28,29]. The gene order of the CTV biosynthesis gene cluster was tremendously similar when comparing *A. terreus* and *P. citreonigrum* (Figure 1), suggesting that the origin of them may be same. This is evolutionally interesting and the function of the genes may be conserved.

On the other hand, the gene that shows high homology to *ctvE* was located on scaffold 19, which is different from the scaffold that *ci-ctvA* to *ci-ctvD* were located on (Figure 1, g2666). Scaffold 16 and 19 can be combined if more precise genome sequencing emerges. However, these genes that are found in the middle of each scaffold imply that *ci-ctvA* to *ci-ctvD* and g2666 do not form a gene cluster in *P. citreonigrum. ctvE* is thought to be a F1-ATPase β-subunit, which is a known target of citreoviridin [16]. Lin et al. [16] demonstrated that *A. terreus* has an extra copy of the F1-ATPase β-subunit, known as *ctvE*, near the CTV biosynthesis cluster, and they predicted that this copy ensures self-resistance against CTV. The genome analysis of the *P. citreonigrum* strain IMI9228 that was performed in this study revealed that there was only one F1-ATPase β-subunit gene (g2666), which is more similar to ATEG_07609, also known as the F1-ATPase β-subunit, rather than *ctvE*. This indicates that the F1-ATPase β-subunit does not duplicate in the *P. citreonigrum* genome. Therefore, *P. citreonigrum* should protect itself from CTV by a different method than by harboring an extra F1-ATPase β-subunit gene. The genome analysis that was performed in this study predicted a gene that was not found in the *A. terreus* genome, specifically between *ci-ctvC* and *ci-ctvD*. This gene is similar to the HC-toxin efflux carrier TOXA and has an MFS_Azr1_MDR_like domain, which is shared among multidrug resistance transporters of the major facilitator superfamily [30]. Efflux-mediated toxin resistance to trichothecenes is well-regarded in certain trichothecene-producing *Fusarium* species [31,32]. *P. citreonigrum* may achieve self-resistance against CTV by possessing an MFS transporter in the biosynthesis cluster of CTV. These results strongly suggest that *ci-ctvA* to *ci-ctvD* are involved in the CTV biosynthesis in *P. citreonigrum*.

To validate the responsibility of the genes for CTV biosynthesis in *P. citreonigrum*, expression analysis was performed (Figure 3). The expression level of *ci-ctvA* was highest at day 4, that is immediately before CTV production peak and then gradually decreased on YES agar (Figure 2 and Figure 3a). Since *ci-ctvA* is believed to be a HR-PKS gene, in which the encoding enzyme accepts acetyl-CoA as the starter unit for CTV and catalyzes iterations of both the malonyl-CoA extension and SAM-dependent methylation at the beginning of CTV biosynthesis [16], the expression profile is correlated with CTV production. On the other hand, the expressions of *ci-ctvB*, *ci-ctvC* and *ci-ctvD* peaked on day 7, which is correlated with CTV production. These three genes operate in the steps of CTV biosynthesis after the *ci-ctvA* step, and their expression profiles correlate with CTV production. Moreover, expressions of all four genes decreased on YES agar with 5% NaCl, whereby CTV production was suppressed. These results ascertain the potential of *ci-ctvA* to *ci-ctvD* as key genes for CTV biosynthesis in *P. citreonigrum*, although further analysis, such as deficient mutant analyses, must be conducted to confirm responsibility for CTV production.

## 4. Conclusions

The draft genome of the *P. citreonigrum* strain IMI92228 was first determined and the genome analysis revealed the presence of a gene cluster quite similar to the CTV biosynthetic gene cluster of *A. terreus*. In the cluster, there were all four genes that were believed to be sufficient for CTV formation in *A. terreus*, and the gene expression profiles were highly correlated with CTV production, which indicate involvement in CTV production in *P. citreonigrum*. Furthermore, it was found that the cluster contains a putative transporter, which may be involved in self-resistance against CTV. Lastly, we conclude that the gene cluster found is a CTV biosynthesis cluster in *P. citreonigrum*. This finding of a gene cluster for CTV in *P. citreonigrum* will expedite efforts towards better understanding of the biosynthetic pathway of CTV, which ultimately leads to the management of CTV contamination in agricultural products, especially rice.

## 5. Materials and Methods

### 5.1. Strain

*Penicillium citreonigrum* strain IMI92228 was obtained from the culture collection of Centre for Agriculture and Bioscience International (CABI) in the United Kingdom and stored on potato dextrose agar (PDA; Eiken, Tokyo, Japan) slant medium.

### 5.2. Whole Genome Sequencing

The *P. citreonigrum* strain IMI92228 was cultured on Potato Dextrose (Difco Laboratories, Franklin Lakes, NJ, USA) liquid medium for 2 days at 25 °C. Genomic DNA was extracted using DNeasy (QIAGEN, Hilden, Germany) according to the manufacturer’s instructions and quantified using a NanoDrop 1000 spectrophotometer (Thermo Fisher Scientific, MA, USA). Library construction and sequencing for the Illumina HiSeq 2500 was provided as a custom service of Eurofins Genomics K. K. (Tokyo, Japan). Genomic DNA samples were sheared to 350 bp fragments by sonication with a sonicator (Covaris, MA, USA). The resulting DNA fragments were processed for adaptor ligation and underwent amplification to generate DNA libraries. Prepared libraries were subjected to paired-end 2 × 100 bp sequencing with the HiSeq 2500 platform (Illumina, San Diego, CA, USA). Obtained raw reads were trimmed and filtered by quality. Each read was trimmed from both ends until all bases in the read surpassed 20 in a Phred quality score. De novo assembly was performed using Platanus 1.2.4 [33] under default settings. The scaffolds that are shorter than 500 bp were discarded, and 80 scaffolds were subjected to the following analysis. For ORF prediction, a web service version of AUGUSTUS (http://bioinf.uni-greifswald.de/webaugustus/prediction/create) [34] was used. The settings of *A. fumigatus* were used as the reference for ORF prediction and the predicted ORFs were subjected to BLAST. The predicted ORFs that aligned to the genes that were involved in the CTV biosynthesis of *A. terreus* were manually checked and identified as the CTV biosynthesis genes in *P. citreonigrum*. The genome completeness of the *P. citreonigrum* strain IMI92228 were estimated by a single copy gene set of *Eurotiomycetes* using the BUSCO pipeline [35].

### 5.3. Citreoviridin Production

*P. citreonigrum* strain IMI92228 was cultured on either YES agar or YES–5% NaCl agar for 14 days at 25 °C. The YES agar had the following formulation: 4 g/L of yeast extract (Difco Laboratories), 20 g/L of sucrose (Mitsui Sugar Co. Ltd., Tokyo, Japan) and 15 g/L of agar (Difco Laboratories) in distilled water. CTV was extracted from the plug with 1 mL of chromophore. After sonication for 20 min, the chloroform layer was collected and dried up under N_2_. The extract was resolved with 1 mL of acetonitrile. Detection and quantification were performed using liquid chromatography–tandem mass spectrometry (LC-MS/MS).

### 5.4. LC–MS/MS Conditions

LC–MS/MS analysis was performed with a Triple Quad 4500 system (AB Sciex, Foster City, CA) equipped with an ESI source and an LC-20A series high-performance LC system (Shimadzu Corp., Kyoto, Japan). The column used was a 150 mm × 2.1 mm i.d., 3 μm, InertSustain C18 (GL Sciences Inc., Tokyo, Japan). The mobile phase was a binary gradient of solvent A (water containing 0.1% formic acid) and solvent B (acetonitrile containing 0.1% formic acid) programmed as follows: at 0 min, 30% B; at 8 min, 80% B; and at 10 min, 80% B. The total run time was 15 min, which included 5 min of equilibration. The flow rate was set at 0.2 mL/min. Chromatographic separation was achieved at 40 °C. The injection volume was 2 μL. The ESI source was operated at 300 °C in the positive ionization mode. Other MS parameters were as follows: curtain gas, 40 psi; ion spray voltage, 5500 V; nebulizer gas (GS1), 30 psi; turbo heater gas (GS2), 80 psi; and collision-activated dissociation gas, 7 (arbitrary units). The multiple reaction monitoring transitions of CTV were 403 [M+H]^+^ to 315 (collision energy, 9 eV) and 403 [M+H]^+^ to 139 (collision energy, 31 eV). The retention time of the CTV in the sample solution (8.8 min) was consistent with that of the analytical standard. The standard of CTV was purchased from FERMENTEK Ltd. (Jerusalem, Israel).

### 5.5. Gene Expression

The *P. citreonigrum* strain IMI92228 was cultured on YES agar or YES–5% NaCl agar for 14 days at 25 °C. The mycelia were stored in RNA*later*^TM^ Stabilization Solution (Thermo Fisher Scientific, Waltham, MA USA) at −80 °C. Mycelia were frozen by liquid nitrogen and clashed using an SK mill (Tokken, inc., Chiba, Japan). Total RNA were isolated using the Maxwell^®^ RSC instrument and SimplyRNA tissue Kit (Promega, Madison, WI, USA), according to the manufacturer’s instructions. RNA quantity and quality were assessed using a NanoDrop 1000 spectrophotometer (Thermo Fisher Scientific). cDNA synthesis was performed using PrimeScript™ RT reagent Kit with gDNA Eraser (Perfect Real Time) (TaKaRa, Shiga, Japan) using 250 ng of total RNA, according to the manufacturer’s instructions.

qPCR was performed with a Thermal Cycler Dice^®^ Real Time System III (TaKaRa) using TB Green^®^ Premix DimerEraser™ (Perfect Real Time) (TaKaRa). Each 20 μl of reaction mixture contained 2 μL of cDNA sample, 10 μL of 2 × TB Green Fast qPCR Mix and 100 nM of each primer. The amplification condition was one cycle for 2 min at 50 °C and 95 °C each, followed by 45 cycles at both 95 °C for 15 s and at 60 °C for 30 s. Melting curve analysis was performed to ensure the specificity of the PCR products. The PCR for each target gene was performed in duplicate for every sample. The data from qPCR were normalized to *β-tubulin* amplification using the comparative threshold cycle (Ct) method [36]. The primers used for amplification are listed in Table 3. The experiments were repeated with three independent cultures. A Student’s *t*-test was used to determine the differences in the expression levels compared to those of a 4-day culture sample on YES agar.

### 5.6. Statistical Analyses

The mean and SD of each value were calculated. Differences between cultures on YES agar and those on YES agar with 5% NaCl were analyzed using a Student’s *t*-test. Statistical *p* values of <0.05 were considered statistically significant.

## Figures and Tables

**Table 1:** The assemble status of *Penicillium citreonigrum* strain IMI92228.

Number of Scaffolds	Minimum Length of Scaffold	Maximum Length of Scaffold	N50	Sum of Length
79	503 bp	2,963,310 bp	1,448,320 bp	27,997,905 bp

**Table 2:** The result of homology search for citreoviridin biosynthesis genes by protein basic local alignment search tool.

Query Gene ^1^		Hit Open Reading Frame (ORF)					E-value	Identity (%)	Coverage (%)
Name	Size (aa)	Number	Size (aa)	Scaffold	Direction	Position in Scaffold			
*ctvA*	2436	g1460	2513	16	+	519,757..527,298	0	70.4	99.9
*ctvB*	228	g1459	233	16	-	518,431..519,132	3E-143	79.9	100
*ctvC*	473	g1456	472	16	-	512,588..514,173	0	77.4	97.9
*ctvD*	354	g1458	341	16	-	517,137..518,229	8E-146	59.9	99.7
*ctvE*	468	g2666	519	19	+	939,425..941,478	0	79.4	95.9

^1^ The citreoviridin biosynthesis genes in *Aspergillus terreus* were used as a query.

**Table 3:** Primers used for q PCR.

Gene	Primer Name	Sequence (5′ to 3′)	Reference
*ctvA*	ctvA_1F	AGCGTGGCATGATTACACCAAACC	this study
	ctvA_1R	CAACGTCGGCCATTGAAGAACCTC	
*ctvB*	ctvB_1F	TGTCTGAGAAAGGCTGCCAATCGTG	this study
	ctvB_1R	CAGCACGTACATCAGCGAGATGGA	
*ctvC*	ctvC_1F	CCAATACCGCCATGGAAGCAG	this study
	ctvC_1R	GCAAGCGCTCGTTCAATCGTATC	
*ctvD*	ctvD_1F	GGCGAATCTCTTGGCAGACATC	this study
	ctvD_1R	CCACAGCAAGAAACCACTCATCC	
*ctvE*	ctvE_1F	GTGACTCCAAGGTGTCTCTG	this study
	ctvE_1R	CAATGAAGAGCAGCACATCC	
*β-* *tubulin*	β-tublinF	CGTGTCGGCGACCAGTTC	[37]
	β-tublinR	CCTCACCAGTGTACCAATGCA	
